# Evaluation of endometrial thickness by transvaginal ultrasound and baseline risk factors as a predictor for endometrial abnormalities in postmenopausal women

**DOI:** 10.1002/ajum.12311

**Published:** 2022-08-21

**Authors:** Jyothirmayi Yerrisani, Anoushka Kothari, Kelly Collins, Emma Ballard, Alka Kothari

**Affiliations:** ^1^ Logan Hospital Meadowbrook Queensland Australia; ^2^ James Cook University Townsville Queensland Australia; ^3^ Northwest Private Hospital Everton Park Queensland Australia; ^4^ The Wesley Hospital Auchenflower Queensland Australia; ^5^ QIMR Berghofer Medical Research Institute Brisbane Queensland Australia; ^6^ Redcliffe Hospital Redcliffe Queensland Australia; ^7^ The University of Queensland St Lucia Queensland Australia

**Keywords:** carcinoma, endometrium, hormone replacement therapy, post‐menopausal, risk factors, transvaginal ultrasound

## Abstract

**Introduction/Purpose:**

To evaluate the endometrial thickness (ET) as a predictor of endometrial abnormalities in postmenopausal women and whether consideration of baseline risk factors increases diagnostic accuracy.

**Methods:**

This is a retrospective observational study of postmenopausal women presenting with bleeding or thickened endometrium (≥4 mm) on ultrasound, between 2003 and 2012. Risk factors for endometrial abnormality were analysed using logistic regression. Of 301 women, 220 were symptomatic and 81 were asymptomatic. The median ET was 6 mm (IQR 4–9) for symptomatic women and 9 mm (IQR 6–12) for asymptomatic women.

**Results:**

Abnormal pathology was found in 35 symptomatic (15.9%) and 6 asymptomatic women (7.4%). For each 1 mm increase in ET, the odds of an abnormal diagnosis increased by 16.3% (95% CI 9.6–23.5) for symptomatic and 19.9% (95% CI 3.1–39.3) for asymptomatic women. The Youden's index method identified an ET threshold of ≥7.1mm for symptomatic and ≥14.5mm for asymptomatic women. In symptomatic women the sensitivity was 88.6% (95% CI 72.3–96.3) and specificity 69.2% (95% CI 61.9–75.6), while in asymptomatic women the sensitivity was 50.0% (95% CI 13.9–86.1) and specificity was 89.3% (95% CI 79.5–95.0). The addition of age in the symptomatic women model reduced the sensitivity (82.9% (95% CI 65.7–92.8)) but increased the specificity (72.4% (95% CI 65.3–78.6)).

**Conclusion:**

ET is a significant predictor of abnormality. In the absence of risk factors, our study suggests that invasive procedures may be withheld until the ET is ≥7.1 mm with bleeding and ≥14.5 mm in asymptomatic women with no bleeding.

## Introduction

Endometrial cancer is the most common gynaecological cancer in women in developed countries.^1^ It presents with postmenopausal bleeding in >90% of cases.^2^ The age‐standardised incidence in Australia is 18.1 per 100,000 women with a 5‐year survival rate of approximately 86%.^3^, ^4^ Menopause is defined retrospectively as the cessation of periods for 12 months.^5^ Therefore, we define symptomatic postmenopausal bleeding as the presence of vaginal bleeding after menopause. Asymptomatic postmenopausal women are defined as those who have not had bleeding but have had an incidental finding of a thickened endometrium of >5 mm on ultrasound.^6^ As the risk of cancer increases by 64‐fold in the presence of postmenopausal bleeding, it warrants timely diagnostic assessment which should be safe, simple and as minimally invasive as possible.^7^, ^8^ Transvaginal sonographic evaluation of the endometrium is the initial investigation of choice, although histopathology remains the gold standard.^9^, ^10^, ^11^ The decision to proceed with invasive testing should be made after careful clinical consideration to avoid complications such as fluid overload and perforation, as well as consideration of the costs associated with these procedures.^9^, ^12^, ^13^, ^14^ It is recommended that biopsy be performed if the endometrial thickness (ET) is >5 mm in women presenting with postmenopausal bleeding and >11 mm in those who are asymptomatic, with no history of postmenopausal bleeding.^2^, ^13^ In their meta‐analysis, Smith‐Bindmann calculated a cancer risk of 6.7% at a cut‐off of 11 mm in asymptomatic women with no history of breast cancer or use of hormone replacement therapy (HRT). The risk of cancer in symptomatic women increases from 0.07% at a thickness of <5 mm to 7.3% at a thickness of >5 mm.^15^ In addition, at an ET of 5 mm, transvaginal ultrasound has a sensitivity and specificity of 96% and 61% respectively in symptomatic women and 77% and 85.8% respectively in asymptomatic women.^16^, ^17^

**Figure 1:**
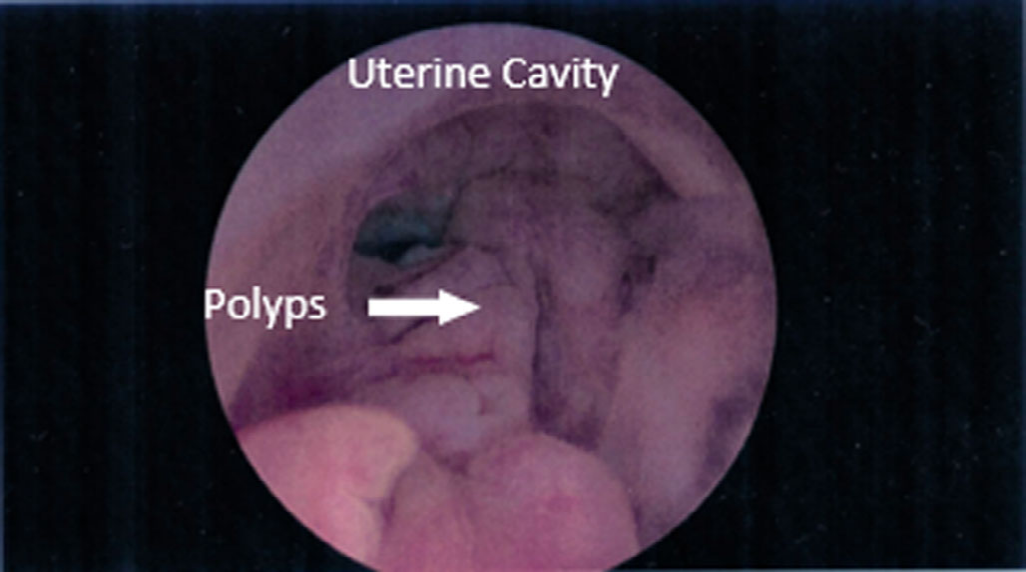
Hysteroscopic image demonstrating multiple benign polyps (arrow) in a female with an endometrial thickening of 7 mm on transvaginal ultrasound. [Colour figure can be viewed at wileyonlinelibrary.com]

Currently, there is insufficient evidence to determine whether transvaginal ultrasonography or endometrial sampling is the most effective method to rule out cancer as the studies are not based on individual risk factors.^18^ Endometrial thickness has a considerable variation in the normal range amongst postmenopausal women and is modified by patient characteristics and risk factors, which in turn influence the diagnostic accuracy of ultrasound.^10^ The endometrium may be uniformly thickened (5–11 mm) in about 10–17% of asymptomatic postmenopausal women or may have focal thickening due to benign conditions, usually polyps (see Figure 1).^9^, ^19^ Universal screening results in a high number of false‐positive results and unnecessary investigations.^20^, ^21^, ^22^, ^23^, ^24^ Consideration of the presence of risk factors such as obesity, hypertension and late menopause should be emphasised before proceeding to invasive diagnostic procedures.^13^, ^24^

**Figure 2:**
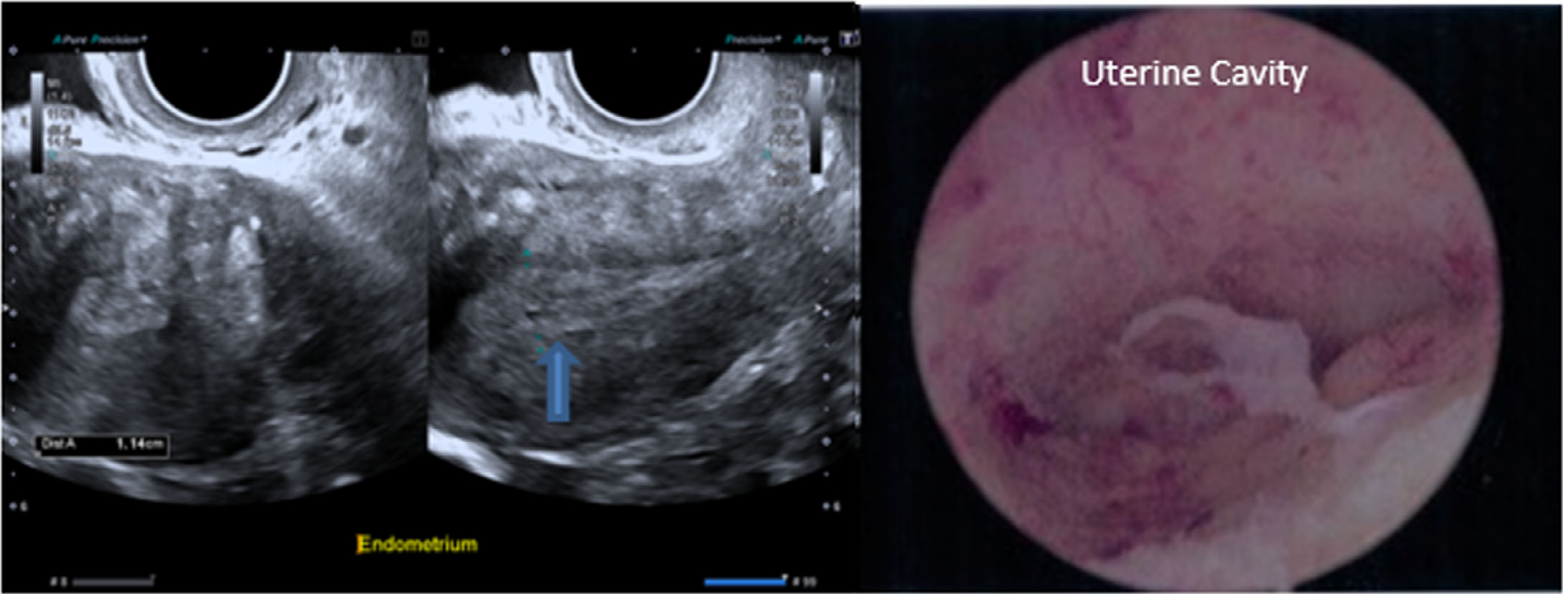
Transvaginal ultrasound and hysteroscopic images for a 52‐year‐old patient, not on hormone replacement therapy, with postmenopausal bleeding and an 11 mm thickened endometrium. Histology confirmed a benign active endometrium. [Colour figure can be viewed at wileyonlinelibrary.com]

**Figure 3:**
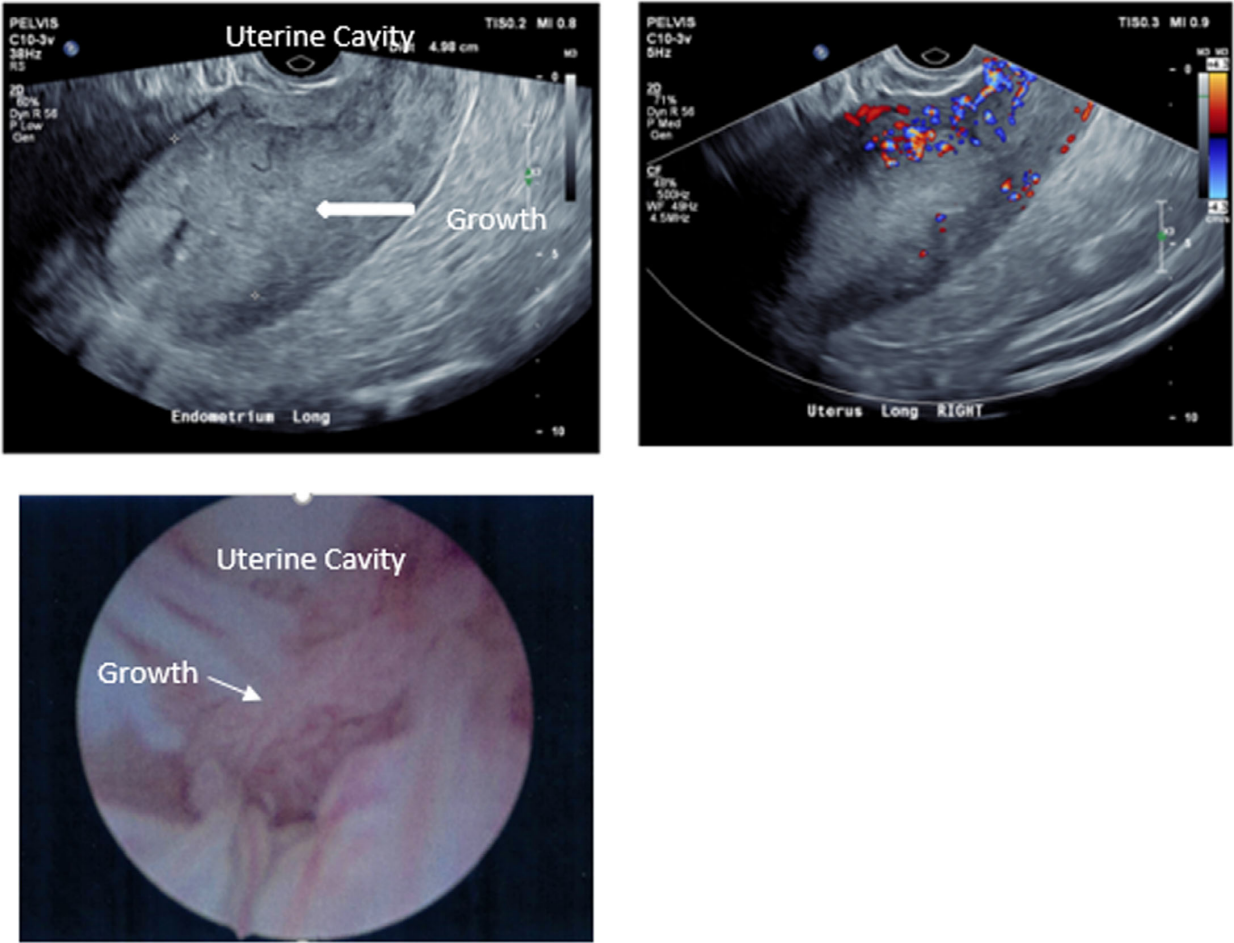
Transvaginal ultrasound and hysteroscopy findings for a 61‐year‐old postmenopausal female not on hormone replacement therapy with a history of bleeding of one month. Risk factors include Type II diabetes, hypertension and hypercholesterolaemia. Endometrium was significantly thickened at 5 cm and highly vascular on ultrasound. Histology showed endometrial adenocarcinoma(arrow). [Colour figure can be viewed at wileyonlinelibrary.com]

Obesity is a modifiable and independent risk factor, which is associated with an increased risk of developing endometrial cancer as well as related mortality (see Figure 2).^25^ The two most important predictors of endometrial cancer are age and years since menopause.^26^, ^27^ Furthermore, diabetes is associated with a two‐fold increase in the risk of endometrial cancer and, in the presence of obesity and hypertension, this risk increases by a further tenfold.^28^ Lynch Syndrome is characterised by an increased risk of bowel, endometrial, ovarian, renal and urinary tract, hepatobiliary and skin malignancies.^29^ The risk of endometrial cancer is substantially increased in the order of 50%, therefore universal screening is recommended in these women.^30^, ^31^ Additionally, in tamoxifen users, the risk of cancer increases 2.4 times and although routine screening is not recommended, it is advised that patients be counselled regarding the risks of therapy.^32^ Analysis from pooled data shows no association between nulliparity and risk of cancer (Figure 3).^33^

The aim of our study was to evaluate the role of ET in predicting abnormal endometrial histopathology in symptomatic and asymptomatic postmenopausal women and to assess whether incorporation of baseline risk factors increases the diagnostic accuracy of transvaginal ultrasound.

## Materials and methods

This was a retrospective observational study conducted at Redcliffe Hospital, an outer metropolitan hospital in Queensland, Australia; over a 10‐year period (January 2003–December 2012). The patient records of all postmenopausal women greater than 50 years of age who had a hysteroscopy for postmenopausal bleeding or who had an incidental finding of asymptomatic thickened endometrium (>4 mm) on ultrasound were reviewed. Human Research Ethics Committee approval and a waiver of consent for the purpose of research under the Public Health Act were obtained from the Prince Charles Hospital Human Research Ethics Committee (HREC 13/QPCH/169). The following variables of interest, which are known risk factors for endometrial carcinoma, were obtained from the patient's records: age, ethnicity, years since menopause, diabetes mellitus, hypertension, polycystic ovarian syndrome, parity, body weight, body mass index (BMI), symptomatic or asymptomatic presentation, use of (HRT), anticoagulants and tamoxifen use, ET on ultrasound, personal or family history of cancer, as well as the results of histopathology and any procedure‐related complications. Histopathology reports were categorised as normal when diagnosed as: inactive, proliferative, atrophic endometrium or benign polyp. Abnormal histology was defined as the presence of simple or complex hyperplasia, with or without atypia, or endometrial cancer.

### Statistical analysis

The statistical analyses were conducted using SPSS version 22 (IBM Corporation, Armonk, New York). Categorical variables were examined using Pearson's chi‐squared test with Fisher's exact test used when more than 20% of the expected values were <5. A Student's *t*‐test or Mann–Whitney U test for skewed data was used to examine continuous variables. Variables significant at the 20% level in univariate analysis were examined using backward elimination in multivariable logistic regression. The odds ratio for abnormal diagnosis and 95% confidence intervals were reported. Youden's index was used to identify the ET which maximised both the sensitivity and specificity for the prediction of endometrial abnormality in symptomatic and asymptomatic women. Youden's index was also used to identify the predicted probability cut‐off from a logistic regression model containing age and ET in symptomatic women. A two‐sided P‐value of <0.05 was considered statistically significant. Area under the curve was reported. Sensitivity, specificity, positive predictive value and negative predictive value as well as their 95% confidence intervals were calculated using the online calculator available at http://vassarstats.net/clin1.html.

## Results

### Study population

A total of 637 records for patients 50 years of age or older were reviewed and characterised into two groups: symptomatic women with bleeding, and asymptomatic women with no bleeding and an incidental finding of a thickened endometrium of >4 mm on ultrasound. Three hundred and thirty‐six patients were excluded due to premenopausal dysfunctional uterine bleeding or previous history of uterine or cervical cancer. Of the 301 postmenopausal women meeting the inclusion criteria, 220 were symptomatic and 81 were asymptomatic.

Ninety‐seven percent of women in this study were Caucasian, the mean age at presentation was 62.8 years (SD 9.3) and 7.5% of women were nulliparous. Twenty‐one percent of women had a history of HRT use and 6.3% had used tamoxifen. Table 1 shows that the median ET in symptomatic women was 6 mm (IQR 4–9) and in asymptomatic women, 9 mm (IQR 6–12).

**Table 1:** Univariate analysis of baseline demographics for the symptomatic and asymptomatic groups

Patient characteristics	Symptomatic women				Asymptomatic women			
	Overall	Normal	Abnormal	p value	Overall	Normal	Abnormal	p value
	n = 220	n = 185	n = 35		n = 81	n = 75	n = 6	
Endometrial thickness, median (IQR)	6 (4–9)	5 (4–8)	12 (8–19)	<0.001	9 (6–12)	9 (6–11)	13 (7–24)	0.071
Age, yr, mean (SD)	61.5 (8.8)	60.1 (7.9)	68.7 (9.9)	<0.001	66.2 (9.8)	66.1 (10.0)	67.7 (8.1)	0.71
Null parity^a^	15 (7.4%)	12 (7.0%)	3 (9.1%)	0.72	6 (7.9%)	6 (8.6%)	0 (0.0%)	1.00
Parity, median (IQR)^a^	3 (2–3)	2 (2–3)	3 (2–4)	0.093	2 (2–4)	2 (2–3)	3 (2–4)	0.56
Weight, mean (SD)^b^	83 (19)	82 (18)	88 (24)	0.12	77 (19)	78 (20)	72 (12)	0.51
BMI, mean (SD)^c^	32.4 (7.7)	32 (7)	35 (9)	0.18	30.6 (7.7)	31 (7.8)	26 (4.3)	0.28
Caucasian ethnicity, n (%)	213 (96.8%)	178 (96.2%)	35 (100.0%)	0.60	78 (96.3%)	72 (96.0%)	6 (100.0%)	1.00
History of tamoxifen/other treatment, n (%)	10 (4.5%)	8 (4.3%)	2 (5.7%)	0.66	9 (11.1%)	8 (10.7%)	1 (16.7%)	0.52
Family history of cancer, n (%)	15 (6.8%)	15 (8.1%)	0 (0.0%)	0.14	13 (16.0%)	12 (16.0%)	1 (16.7%)	1.00
Hormone replacement therapy use, n (%)	50 (22.7%)	46 (24.9%)	4 (11.4%)	0.082	13 (16.0%)	12 (16.0%)	1 (16.7%)	1.00
Anticoagulant use, n (%)	36 (16.4%)	24 (13.3%)	12 (34.3%)	0.002	21 (26.8%)	21 (28.0%)	0 (0.0%)	0.33
Diabetes, n (%)	36 (16.4%)	27 (14.6%)	9 (25.7%)	0.10	15 (18.5%)	15 (20.0%)	0 (0.0%)	0.59
Hypertension, n (%)	100 (45.5%)	79 (42.7%)	21 (60.0%)	0.059	38 (46.9%)	35 (46.7%)	3 (50.0%)	1.00
Personal history of cancer, n (%)	9 (4.1%)	7 (3.8%)	2 (5.7%)	0.64	22 (27.2%)	21 (28.0%	1 (16.7%)	1.00
Menopause, n (%)^d^				<0.001				0.58
<5	92 (44.7%)	89 (50.6%)	3 (10.0%)		20 (30.8%)	19 (32.2%)	1 (16.7%)	
6–10	40 (19.4%)	32 (18.2%)	8 (26.7%)		8 (12.3%)	7 (11.9%)	1 (16.7%)	
11–15	21 (10.2%)	16 (9.1%)	5 (16.7%)		8 (12.3%)	8 (13.6%)	0 (0.0%)	
16–20	19 (9.2%)	15 (8.5%)	4 (13.3%)		9 (13.8%)	7 (11.9%)	2 (33.3%)	
>20	34 (16.5%)	24 (13.6%)	10 (33.3%)		20 (30.8%)	18 (30.5%)	2 (33.3%)	

BMI, body mass index.

^a^ Symptomatic n = 204, asymptomatic n = 76.

^b^ Symptomatic n = 217.

^c^ Symptomatic n = 120, asymptomatic n = 40.

^d^ Symptomatic n = 206, asymptomatic n = 65.

Endometrial thickness, age, parity and weight (BMI, family history of cancer, use of HRT, use of anticoagulants, diabetes, hypertension and years of menopause) were considered possible predictors of an abnormal endometrial diagnosis during univariate analysis for symptomatic women and were further screened using logistic regression. ET and use of anticoagulants were identified as possible predictors of an abnormal diagnosis during univariate analysis for asymptomatic women. Anticoagulants were not used in those with an abnormal result and so only ET was used as a predictor in the logistic regression for this group. The final models for both set of women are shown in Table 2.

**Table 2:** Logistic regression models predicting an abnormal diagnosis in symptomatic and asymptomatic women

Model	Variables	Odds ratio (95% CI)	p value
Symptomatic women model			
Univariate model	Endometrium thickness	1.163 (1.096–1.235)	<0.001
Adjusted model	Endometrium thickness	1.158 (1.086–1.234)	<0.001
	Age	1.100 (1.051–1.152)	<0.001
Asymptomatic women model			
Univariate model	Endometrium thickness	1.199 (1.031–1.393)	0.018

Endometrial thickness was an important predictor of endometrial abnormality in both symptomatic and asymptomatic women. For each 1 mm increase in ET, the odds of having an abnormal diagnosis increased by 16.3% (95% CI 9.6–23.5) for symptomatic women and by 19.9% (95% CI 3.1–39.3) for asymptomatic women. Age was also identified as an important predictor in symptomatic women with the odds of having an abnormal diagnosis increasing by 10.0% (95% CI 5.1–15.2) with each year.

The Youden's index method was used to identify an ET threshold of ≥7.1 mm for symptomatic patients, which correctly identified 31/35 abnormal and incorrectly identified 57/185 normal endometrial pathology, resulting in a sensitivity of 88.6% (95% CI 72.3–96.3) and specificity of 69.2% (95% CI 61.9–75.6), as shown in Table 3. In contrast, using a predicted probability cut‐off of 0.117 after additionally adjusting for age, correctly identified 29/35 abnormal and 51/185 cases with normal endometrial pathology, resulting in a sensitivity of 82.9% (95% CI 65.7–92.8) and specificity of 72.4% (95% CI 65.3–78.6). A cut‐off of ≥14.5 mm was identified using the Youden's index method for asymptomatic women correctly identifying 3/6 abnormal cases and incorrectly identifying 8/75 normal cases of endometrial pathology, resulting in a sensitivity of 50.0% (95% CI 13.9–86.1) and specificity of 89.3% (95% CI 79.5–95.0).

**Table 3:** Diagnostic summary for each cut‐off in symptomatic and asymptomatic women

Parameter	Symptomatic women		Asymptomatic women
	Endometrium thickness only with cut‐off of ≥7.1 mm	Endometrial thickness and age with a predicted probability cut‐off ≥0.117	Endometrium thickness only with cut‐off ≥14.5 mm
	Value (95% CI)	Value (95% CI)	Value (95% CI)
AUC	0.825 (0.758–0.891)	0.841 (0.770–0.911)	0.722 (0.494–0.950)
Sensitivity (%)	88.6 (72.3–96.3)	82.9 (65.7–92.8)	50.0 (13.9–86.1)
Specificity (%)	69.2 (61.9–75.6)	72.4 (65.3–78.6)	89.3 (79.5–95.0)
PPV (%)	35.2 (25.5–46.2)	36.3 (26.0–47.8)	27.3 (7.3–60.7)
NPV (%)	97.0 (91.9–99.0)	95.7 (90.5–98.2)	95.7 (87.2–98.9)

AUC, Area under the curve; PPV, Positive Predictive Value; NPV, Negative Predictive Value.

In, symptomatic women, if a cut‐off of 4 mm was used, which is commonly utilized in the literature, there would have been no false negatives but there would have been an 80% false positive rate. While a cut‐off of 7.1 mm has a 3% false negative rate it has a 65% false positive rate, sparing significantly more women from more invasive tests. There were 185 (84.1%) women in the symptomatic group with normal pathology. Of these, 134 were found to have atrophy, 49 had polyps and two had warfarin‐induced bleeding. There were 35 (15.9%) women in the symptomatic group with an abnormal ET. Of these women, 24 had endometrial carcinoma, five had simple hyperplasia, four had complex hyperplasia with atypia, one had cervical cancer and there was one case of leiomyosarcoma. There were 75 (92.6%) women in the asymptomatic group with normal findings on histopathology; 39 were found to have atrophy and 36 had polyps. For the asymptomatic women, six (7.4%) had an abnormal endometrial pathology. Of these, three had simple hyperplasia, two had endometrial carcinoma and one had simple hyperplasia with atypia.

## Discussion

In this study, we evaluated whether considering the presence of potential risk factors for endometrial cancer increased the diagnostic accuracy of transvaginal ultrasound. We hypothesised that the combination of risk factors along with ET measurement from ultrasound would increase the sensitivity and specificity to detect endometrial abnormality. ET was found to be a significant predictor of endometrial pathology. In the presence of endometrial pathology, the median thickness was high in both symptomatic and asymptomatic groups. This measurement was 12 mm (IQR 8–19 mm) in the symptomartic group and 13 mm (IQR 7–24 mm) in the asymptomatic group, suggesting a direct association with endometrial abnormality, as noted by previous studies.^7^, ^10^, ^34^, ^35^, ^36^, ^37^

In the symptomatic group, a cut‐off of 7.1 mm to detect endometrial abnormality gave a sensitivity of 88.6% and specificity of 69.2%. This cut‐off, although higher than what is typically recommended, is consistent with the study of Mateaos *et al.,* ^38^ who suggest that endometrial sampling should be avoided if ET is ≤6 mm. In our study, no abnormal pathology was identified at a cut‐off of <4 mm, which is similar to previously published literature.^7^, ^39^, ^40^ A cut‐off of 7.1 mm for symptomatic women would have missed four abnormal cases but spared 128 of the 185 normal cases from undergoing an invasive procedure. When age was also taken into consideration, six cases with an abnormality would have been missed, however, 134 cases with no pathology would have been spared a hysteroscopy. This suggests that invasive procedures can potentially be deferred if the ET is <4 mm.^12^, ^13^, ^15^, ^38^, ^39^ Additionally, in the symptomatic group, age of presentation and years since menopause were significant predictors at the univariate level but were not found to be significant with multivariable modelling. These factors have been previously identified in studies by Bruchim, Tsuda and Salman *et al*.^27^, ^41^, ^42^ In the asymptomatic group, a cut of 14.5 mm to detect endometrial pathology had a sensitivity of 50.0% and a specificity of 89.3%. Similar findings were published by Soha Siam at a cut‐off of 12 mm.^43^

Risk factors such as obesity, diabetes, hypertension, tamoxifen and polycystic ovary syndrome are currently being used to determine whether invasive biopsies should be taken.^44^ However, our study did not identify these risk factors at the univariate level. Weight and BMI, where available, were higher in patients with abnormal pathology, however these differences were not statistically significant. This might be secondary to incomplete reporting of the variable of BMI in almost 50% of our patient records (120/220 and 40/81) and a small sample size. Reproductive factors and HRT were not associated with an abnormal finding, which is consistent with one study suggesting that nulliparity and HRT use had no hormone‐dependent increase in the risk for cancer, but at odds with a recent review which purports an increase in the risk of endometrial cancer with hormone therapy.^35^, ^37^, ^45^ The major and minor complication risks with hysteroscopy were 0.5% and 3.7% in this study, which was similar to those cited by Lev‐Sagie.^46^ Although the complication rate is low, it is still very important to consider the individual clinical criteria to avoid unnecessary anxiety, pain and complications and to improve the cost‐effectiveness of investigations.^13^, ^47^ Even though transvaginal endometrial evaluation is the primary investigation of choice to predict endometrial pathology in postmenopausal women, its sensitivity is still controversial as it is highly variable amongst women. This study demonstrates that ET is significantly more important than any of the other baseline risk factors examined for both symptomatic and asymptomatic women. Adjusting for age did not significantly improve sensitivity and specificity in symptomatic women. The authors suggest that the proposed model for asymptomatic women be used with caution due to the small number of cases with an abnormal diagnosis in this dataset.

## Limitations

As this was a retrospective study, there may have been unmeasured variables that influenced the outcome. There were a small number of abnormal findings in the study sample, and this is reflected in the width of the confidence intervals. As majority of the patients included in the study were Caucasian, the findings may not be applicable to more ethnically diverse populations. We did not have BMI recorded in a significant number of medical records. We also excluded cases with an ET of <4 mm in asymptomatic women, as the pre‐test probability for cancer is low in this patient population. Conclusions regarding the cut‐off of ET in asymptomatic women should only be considered as a guide for the development of future studies. Prospective and multicentric studies are needed to evaluate ultrasound measurements of ET and to further evaluate the role of risk factors in the detection of endometrial cancer.

## Conclusion

Endometrial thickness is a significant predictor of endometrial abnormality in both symptomatic and asymptomatic postmenopausal women. Of all the risk factors examined, only age was found to be an important predictor of endometrial abnormality in the symptomatic group, although the sensitivity was reduced. The authors suggest that in the absence of risk factors or compelling reasons to offer invasive investigations, such procedures may be withheld for postmenopausal women until the ET is ≥7.1 mm when presenting with bleeding and potentially ≥14.5 mm in asymptomatic patients. Further research is required in the setting of risk factors.

## Author contributions

**Jyothirmayi Yerrisani:** Conceptualization (equal); data curation (equal); formal analysis (equal); investigation (equal); methodology (equal); writing – original draft (equal); writing – review and editing (equal). **Kelly Collins:** Writing – original draft (equal); Writing (reviewing and editing (equal). **Anoushka Kothari:** Writing – original draft (supporting); writing – review and editing (supporting). **Emma Ballard:** Formal analysis (lead); writing – original draft (supporting); writing – review and editing (supporting). **Alka Kothari**: Conceptualisation (equal); investigation (equal); methodology (equal); supervision of data collection (lead) investigation (equal); supervision of manuscript writing and editing for final version (lead).

## Consent

Consent was obtained from all patients whose deidentified images have been included in the manuscript.

## Authorship statement

All persons who meet authorship criteria are listed as authors, and all authors certify that they have participated sufficiently in the work to take public responsibility for the content, including participation in the concept, design, analysis, writing or revision of the manuscript. Furthermore, each author certifies that this material or similar material has not been and will not be submitted to or published in any other publication before its appearance in the Australasian Journal of Ultrasound in Medicine.

## Funding

No funding information is provided.

## Conflict of interest

The authors have no disclosures to make, and no funding was received aiding the production of this manuscript. This manuscript has not been submitted nor is it under consideration for publication in any other forum. This study was done for the purpose of a RANZCOG trainee research project for Jyothirmayi Yerrisani.
